# Thyroid disease in ageing populations

**DOI:** 10.1530/EC-25-0393

**Published:** 2025-08-05

**Authors:** Xin Hou, Xiaomeng Sun, Shuhang Xu, Yushu Li

**Affiliations:** ^1^ Department of Geriatrics, The First Affiliated Hospital of China Medical University, Shenyang, Liaoning Province, P.R. China; ^2^ Department of Endocrinology, The Affiliated Hospital of Integrated Traditional Chinese and Western Medicine, Nanjing University of Chinese Medicine, Nanjing, China; ^3^ Jiangsu Province Academy of Traditional Chinese Medicine, Nanjing, China; ^4^ Department of Endocrinology and Metabolism, Institute of Endocrinology, NHC Key Laboratory of Diagnosis and Treatment of Thyroid Diseases, The First Affiliated Hospital of China Medical University, Shenyang, P.R. China

**Keywords:** older adults, thyroid disorders, subclinical hypothyroidism (SCH), thyroid nodules

As the global population ages, the incidence of age-related diseases is steadily increasing, placing a growing economic and healthcare burden on society. Thyroid diseases are highly prevalent among older adults. Data from the Thyroid, Iodine, and Diabetes Epidemiological survey (TIDE survey) indicate that the prevalence of thyroid diseases in this population is significantly higher than in the general adult population, with both hypothyroidism and thyroid nodules showing a clear age-related increase in prevalence (1).

Thyroid hormones play a crucial role in development, growth, and energy metabolism. With advancing age, thyroid hormone production progressively decreases. However, compensatory reductions in hormone clearance maintain serum free thyroxine (FT_4_) concentrations within normal limits, while thyroid-stimulating hormone (TSH) levels become elevated – a biochemical state termed subclinical hypothyroidism (SCH). A longitudinal study found that 60.8% of older adults (≥65 years) with SCH experienced normalization of thyroid function without treatment after a median follow-up of 344 days. Among those with persistent SCH (defined as at least two elevated TSH measurements more than 3 months apart), TSH levels remained normalized in 39.9% after 1 year (2). The clinical management of persistent SCH remains controversial, particularly regarding two key aspects: i) its potential association with increased cardiovascular risk and ii) the uncertain therapeutic benefits of levothyroxine replacement therapy in this population. A systematic review by Yamamoto *et al.* found no significant association between SCH and cardiovascular disease (CVD) incidence, CVD-related mortality, or all-cause mortality in older adults (≥65 years) (3). Given the heterogeneity in age distributions and TSH levels across studies, it remains uncertain whether levothyroxine therapy ameliorates CVD risk factors, CVD events, or CVD mortality in this population (3). Future studies employing age-specific TSH reference ranges are needed to elucidate the SCH–CVD relationship and determine the efficacy of levothyroxine treatment in older adults with SCH (4).

Numerous studies demonstrate that TSH levels exhibit an age-dependent elevation in older adults, highlighting the clinical necessity of establishing age-specific reference ranges to improve diagnostic and therapeutic accuracy (5). In 2020, the French Society of Endocrinology (SFE) established the first international expert consensus for managing thyroid dysfunction in older adults (6). The guidelines propose i) a stable TSH lower reference limit of 0.4 mIU/L across all age groups and ii) an age-adjusted upper limit calculated by dividing the patient’s age by 10 (in years). For example, 7 mIU/L is used for patients aged 70–79 years and 8 mIU/L for octogenarians (≥80 years). Zhai *et al.* emphasized the critical need for stringent exclusion criteria when establishing TSH reference ranges (7). In addition to conventional factors (genetic predisposition, thyroid disease history, iodine status, medication use, and assay methodology), emerging evidence suggests that sleep patterns and chronic diseases associated with thyroid dysfunction may significantly influence TSH levels. For example, Xin *et al.* reported elevated prevalence of thyroid disorders in Chinese sarcoidosis patients (8). Nevertheless, current studies remain limited by small sample sizes and substantial heterogeneity; further research is needed to refine TSH reference ranges for older adults.

Thyroid nodules demonstrate remarkably high prevalence among elderly populations, affecting 68.24% of Chinese adults aged ≥60 years, and their incidence increases with age (1). Although thyroid cancer occurs less frequently in older adults, this population demonstrates distinct pathological characteristics compared to younger patients, including poor differentiation, higher lymph node metastasis rates, advanced tumour stages, and increased recurrence and mortality. Furthermore, the clinical management of older adults with thyroid cancer is complicated by frequent multimorbidity and age-related declines in functional and cognitive capacity. Beyond oncological outcomes, particular attention must be paid to quality-of-life considerations and the psychological impact of both diagnosis and treatment in older adults (9). While surgical resection remains the primary treatment for thyroid cancer, advancing age correlates with poorer prognostic outcomes. A meta-analysis evaluating age-related impacts on total thyroidectomy revealed that patients ≥60 years experienced shorter disease-free survival, reduced overall survival, higher rates of recurrence and metastasis, and increased postoperative complications compared to younger patients (10). Furthermore, Maki *et al.* identified cancer-related fatigue in 41.8% of postoperative papillary thyroid carcinoma patients, with this debilitating symptom significantly compromising quality of life. Notably, serum free triiodothyronine (FT_3_) levels demonstrate a significant association with fatigue severity (9). These findings underscore the necessity for comprehensive risk–benefit analysis when making clinical decisions regarding thyroid nodule management in elderly patients.

The management of thyroid diseases in older adults presents clinical challenges. Age-related alterations in the hypothalamic–pituitary–thyroid axis and progressive multi-system functional decline increase both disease susceptibility and mortality risk. These factors necessitate a specialized approach to geriatric thyroid care. A comprehensive understanding of age-specific disease characteristics, coupled with early detection and thorough clinical assessment, is critical for implementing standardized management protocols. Such an approach not only optimizes clinical outcomes but also reduces healthcare utilization burdens while maintaining quality of life in the ageing population.

## Declaration of interest

The authors declare that there is no conflict of interest that could be perceived as prejudicing the impartiality of this work.

## Funding

This work was funded by the Science and Technology Project for the High-Quality Development of China Medical University, with support from the Department of Biotechnology Development and Industrialization (grant 2023JH2/20200110).
